# Dr. George Henry De Nike

**Published:** 1913-08

**Authors:** 


Dr. George Henry De Nike, Queen's University of Kingston, Ont., 1882, died suddenly in his sanitarium at Clinton, N. Y., June 13, aged about 57.

